# A Novel Automatic Method to Estimate Visual Acuity and Analyze the Retinal Vasculature in Retinal Vein Occlusion Using Swept Source Optical Coherence Tomography Angiography

**DOI:** 10.3390/jcm8101515

**Published:** 2019-09-20

**Authors:** Marta Díez-Sotelo, Macarena Díaz, Maximino Abraldes, Francisco Gómez-Ulla, Manuel G. Penedo, Marcos Ortega

**Affiliations:** 1 Department of Ophthalmology, Complejo Hospitalario Universitario de Santiago de Compostela, 15706 Santiago de Compostela, Spain; 2 Department of Surgery, University of Santiago de Compostela, 15705 Santiago de Compostela, Spain; 3 Department of Computer Science, University of A Coruña, 15071 A Coruña, Spain; 4 CITIC-Research Center of Information and Communication Technologies, University of A Coruña, 15071 A Coruña, Spain; 5 Instituto Oftalmológico Gómez-Ulla, 15706 Santiago de Compostela, Spain

**Keywords:** optical coherence tomography angiography, retinal vein occlusion, automatic method, visual acuity estimation, imaging retina

## Abstract

The assessment of vascular biomarkers and their correlation with visual acuity is one of the most important issues in the diagnosis and follow-up of retinal vein occlusions (RVOs). The high workloads of clinical practice make it necessary to have a fast, objective, and automatic method to analyze image features and correlate them with visual function. The aim of this study is to propose a fully automatic system which is capable of estimating visual acuity (VA) in RVO eyes, based only on information obtained from macular optical coherence tomography angiography (OCTA) images. We also propose an automatic methodology to rapidly measure the foveal avascular zone (FAZ) area and the vascular density (VD) in the superficial and deep capillary plexuses in swept-source OCTA images centered on the fovea. The proposed methodology is validated using a representative sample of 133 visits of 50 RVO patients. Our methodology estimates VA with very high precision and is even more accurate when we integrate depth information, providing a high correlation index of 0.869 with the real VA, which outperforms the correlation index of 0.855 obtained when estimating VA from the data obtained by the semiautomatic existing method. In conclusion, the proposed method is the first computational system able to estimate VA in RVO, with the additional benefits of being automatic, less time-consuming, objective and more accurate. Furthermore, the proposed method is able to integrate depth information, a feature which is lacking in the existing method.

## 1. Introduction 

Retinal vein occlusion (RVO) is the most common cause of the retinal vascular disease after diabetic retinopathy [1], with a prevalence of 5.2 per 1000 people and affecting approximately 16 million people worldwide [2]. Macular edema (ME) is the principal cause of visual loss, which may also result from macular ischemia or neovascularization [3]. 

Recent technological advances in the biomedical field, specifically in the field of ophthalmology, have given us a new perspective on our interpretation of ocular diseases. 

Fluorescein angiography (FA) has been the gold standard for the visualization of vascular features in retinal vascular diseases, until a few years ago when a novel technique based on optical coherence tomography (OCT) technology was introduced to complement existing imaging tools: optical coherence tomography angiography (OCTA). The substantial workload that exists in clinical practice nowadays makes it desirable to speed up the workflow while maintaining quality and integrity. In addition to its demonstrated superiority in assessing macular vascularization, OCTA also has the advantages of being noninvasive and less time-consuming, as compared to the classic technique of FA [4]. Furthermore, this new tool provides stratigraphic vascular details that have not previously been observed by standard fluorescein angiography, allowing us to visualize not only the superficial but also the deep capillary plexus (SCP and DCP, respectively) and the choriocapillaris, so that we can better understand the pathophysiology and define the extent of retinal vascular injuries.

OCTA technology is based on the detection of erythrocytes moving in the blood vessel lumen above a detection rate threshold. It uses different algorithms to measure changes in the reflected OCT signal between two or more consecutive cross-sectional B-scans obtained from a selected region, generating corresponding high-quality angiograms. Swept-source OCTA (SS-OCTA) technology is a depth-resolved imaging modality that improves penetration in ocular tissues such as the retina and choroid, as well as optical opacities and, therefore, does not need enhanced-depth imaging (EDI) techniques to visualize the deepest structures, such as the choroid [5]. SS-OCTA is superior to spectral-domain OCTA (SD-OCTA) when analyzing vascular information from deeper tissues [6].

The DRI SS-OCTA Triton (Topcon Corporation, Tokyo, Japan) allows us to assess the different vascular parameters in OCTA images which have been proposed as suitable biomarkers for severity or visual prognosis in RVO eyes, such as the foveal avascular zone (FAZ) and the vascular density (VD). Although the size of the FAZ in healthy eyes does not seem to influence visual function [7], the area of the FAZ in RVO eyes has been demonstrated to be correlated with visual prognosis [8,9,10]. Furthermore, the VD in the macula has been shown to be another determining parameter in estimating the visual outcome in RVO eyes [10,11,12,13]. The previously demonstrated main damage of the deep capillary plexus induced by retinal vein occlusion [14,15,16,17] makes it necessary to obtain independent information about these parameters in the different capillary plexuses. 

Topcon’s IMAGEnet^®^ software (Topcon Corporation, Tokyo, Japan) offers a tool for manually outlining and measuring the area of the FAZ. This lengthens and introduces subjectivity into the procedure. It also provides semiautomatic quantitative information about vascular density in the 3 × 3 and 6 × 6 mm images of the SCP, but unfortunately does not provide any quantitative information about the DCP. Recently, Díaz et al. [18] validated an automated method for localizing and measuring the FAZ region using 213 healthy and diabetic retinopathy SS-OCTA images.

We propose a novel automatic system to estimate the visual acuity (VA) of RVO eyes, using the only information of 3 × 3 and 6 × 6 mm macular OCTA images. Furthermore, we propose a new, completely automated methodology to rapidly localize, outline, and measure the FAZ area and the vascular density (VD) in the superficial and deep capillary plexus in SS-OCTA 3 × 3 and 6 × 6 mm images centered on the fovea of RVO eyes, introducing automatic quantitative analysis of the deep capillary plexus. We developed two different experiments to assess the validity of the proposed methodology and to demonstrate if the OCTA information of the superficial and deep capillary plexus is sufficient for accurate estimation of the visual acuity in patients diagnosed with RVO.

## 2. Materials and Methods

This prospective study was performed at the Ophthalmology Department of the Clinical University Hospital of Santiago de Compostela, Spain. The study adhered to the tenets of the Declaration of Helsinki and was approved by the “Research Ethics Committee of the Autonomous Community of Galicia (CAEIC)” under the protocol code MAL-OCTA-2018-01. All subjects signed a written informed consent before participating in the study.

### 2.1. Subjects

Patients clinically diagnosed with RVO with macular involvement, of age greater than or equal to 18 years, treatment-naïve, with an absence of refractive error greater than six diopters, and having enough transparency of ocular media to allow quality OCTA images to be obtained were enrolled in this study.

The exclusion criteria were having other retinal diseases and poor quality of obtained OCTA images (signal strength index lower than 40). 

Fifty eyes of 50 patients with naïve RVO (9 patients with central RVO and 41 patients with branch RVO) met the selection criteria, and these patients were recruited for the study. 

All patients underwent three complete ophthalmic examination visits: at baseline, and at 3rd and 6th month follow-ups, including best-corrected visual acuity (BCVA) measurement with an Early Treatment Diabetic Retinopathy Study (ETDRS) chart, funduscopic study, and SS-OCT and SS-OCTA using the swept-source OCTA Triton (Topcon Corporation, Tokyo, Japan) performing 3 × 3 and 6 × 6 mm captures centered on the fovea. To reduce the various types of biases, baseline and follow-up examinations were performed in the morning between 9 and 11 a.m. by the same physician. Antivascular endothelial growth factor (antiVEGF) was the first-line treatment option for those patients who presented ME, receiving an initial loading dose of three monthly intravitreal injections of ranibizumab or aflibercept, following by a pro re nata regimen over a 6-month period. 

Finally, 133 visits (39 at baseline, 47 at 3 months, and 47 at the 6-month follow-up) of 50 RVO patients were included for subsequent analysis, as explained in Results.

### 2.2. Image Acquisition 

The swept-source OCTA Triton (Topcon Corporation, Tokyo, Japan) was used to obtain optical coherence tomography angiography images. This instrument has an A-scan rate of 100,000 scans per second and uses full-spectrum ratio-based amplitude ratio analysis (OCTARA©) detection software (Topcon Corporation, Tokyo, Japan), which has been shown to be more sensitive than other OCTA detection methods in detecting low or slow blood flow in the retina, and it is the only commercially available SS-OCTA device with a 1050 nm light source [5]. This device segments the superficial slab with an inner boundary at 2.6 μm below the top of the internal limiting membrane (ILM) and an outer boundary at 15.6 μm below the inner plexiform layer (IPL), and the deep slab with an inner boundary at 15.6 μm below the IPL and an outer boundary at 70.2 μm below the IPL [19]. 

For each RVO eye in each of the 133 visits, 3 × 3 and 6 × 6 mm scans centered on the fovea were acquired. Automated segmentation of the OCT retinal layers was conducted using Triton software. The 3 × 3 mm images were used to locate and measure the area of the FAZ, and 3 × 3 and 6 × 6 mm images were used to analyze the VD of the superficial and deep capillary plexus. An ETDRS grid with a 1 mm diameter inner ring and 3 mm diameter outer ring was used to center the FAZ and measure the foveal and parafoveal superficial and deep vascular densities.

### 2.3. Manual and Semiautomatic Analysis of the OCTA Images Using Triton Software

The FAZ in the 3 × 3 mm images of the SCP was manually outlined by an experienced investigator (MDS) and the FAZ area was calculated using the Triton software. Vascular density was calculated by the device as the percentage of the area occupied by blood vessels with flow above a threshold detection level in a selected area and depth of vessels. The VD of the SCP, automatically generated by the instrument, was recorded. In the complex (but frequent) RVO cases with macular ischemia, the software of the device did not correctly center the ETDRS grid on the fovea, due to incorrect FAZ identification, and manual correction was necessary. In those cases, we obtained a semiautomatic superficial VD from the instrument.

### 2.4. Automatic Analysis of the OCTA Images Using the Proposed Methodology 

Recently, Díaz et al. [18] validated an automated computational methodology to localize and measure the FAZ region using 213 healthy and diabetic retinopathy SS-OCTA images captured with the DRI OCTA Triton. The principal steps of the methodology are image processing, vascular edge identification, extraction of the FAZ candidates, FAZ region final identification (especially important for pathological conditions, the morphological characteristics of the FAZ are analyzed to remove the main false positives), and precise FAZ segmentation and area calculation.

We based the current study on this validated innovative technology to automatically and successfully extract the FAZ area in the 133 3 × 3 mm OCTA images of the SCP.

**Figure 1:**
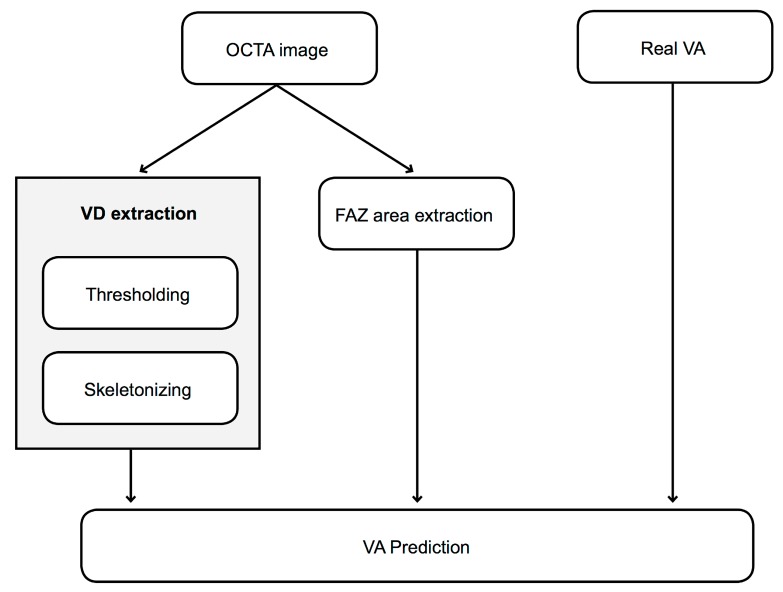
Outline of the main steps of the proposed methodology. OCTA: optical coherence tomography angiography, FAZ: foveal avascular zone, VD: vascular density, VA: visual acuity.

Once the method automatically localizes and extracts the FAZ from the image, it has the capacity to directly calculate information about the foveal and parafoveal VD. We propose a three-step computational methodology based on digital image processing for fully automatic extraction of accurate VD data from the 3 × 3 and 6 × 6 mm OCTA images in the SCP and DCP. The steps of the proposed method are explained in the following points and summarized in Figure 1.

#### 2.4.1. Thresholding Using the OTSU Algorithm

The thresholding method is a pixel-oriented segmentation method. The OCTA images are pixel array representations in grayscale. OTSU is an algorithm that automatically calculates a threshold from a gray level histogram in a nonparametric way, and is derived from the viewpoint of discriminant analysis [20]. The objective is to keep the dispersion in each segment as small as possible while simultaneously keeping the dispersion between different segments as large as possible. This method is based on the following algorithm:

(1) $$Q\left(t\right)=\;\frac{\sigma_{zw}^{2}\left(t\right)}{\sigma_{in}^{2}\left(t\right)},$$

where *Q(t)* is the threshold value that optimizes the segments in terms of variance, *σ^2^_zw_(t)* is the variance between the segments, and *σ^2^_in_(t)* is the variance within the segments. The objective is to segment the pixels of the image and obtain a binary image in black and white (composed of pixels with values 0 and 1). 

#### 2.4.2. Skeletonizing 

This step consists of applying a parallel thinning algorithm for extracting the skeleton of an image by removing all of the contour points of the picture except those that belong to the skeleton, such that only the distinctive features from the pattern remain [21], that is, to extract an image which is only the white skeleton of the image obtained after the thresholding step.

#### 2.4.3. Calculation of the Vascular Density

We finally apply an algorithm using the values of the pixels of the skeletonized image. As the initial OCTA image is a matrix of pixels with values between 0 and 1, and the skeletonized image after thresholding extracts the pixels with a value of 1 (considered the vascular component of the image), we calculate the vascular density as the sum of the values of the pixels divided by the total number of pixels of the skeletonized image. The following algorithm summarizes this procedure:

(2) $$Dens=\;\frac{\sum_{i=0}^{height}\sum_{j=0}^{width}I_{i,j}}{height\;\times \;width},$$

where *I_i,j_* is the value of the pixel.

**Figure 2:**
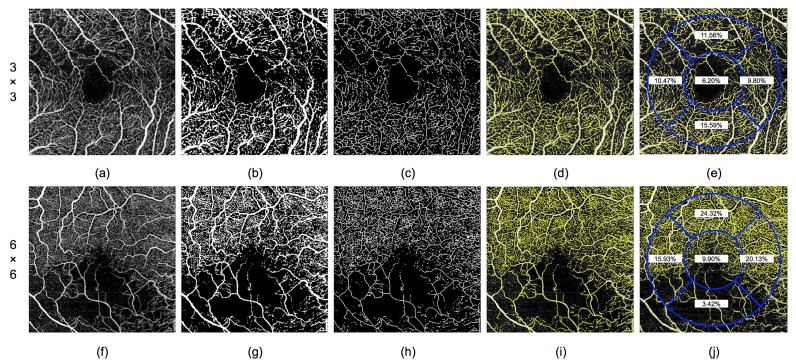
Steps of the automatic analysis of the optical coherence tomography angiography (OCTA) 3 × 3 and 6 × 6 mm images (**a**–**e** and **f**–**j**, respectively) using the proposed methodology. (**a,f**) Original OCTA image centered on the fovea. (**b,g**) Thresholding. (**c,h**) Skeletonization. (**d,i**) Overlap. (**e,j**) Vascular density calculation for each of the five zones of the Early Treatment Diabetic Retinopathy Study (ETDRS) grid, representing the foveal and parafoveal area.

As the areas used for vascular density quantification in this study were the fovea and parafovea, the value of the density was calculated for each of the five zones of the ETDRS grid (foveal, upper, lower, nasal, and temporal) in the 3 × 3 and 6 × 6 mm OCTA images of the superficial and deep capillary plexus (Figure 2).

Finally, a support vector machine (SVM) is used to make the VA estimation based on the extracted parameters. SVM is a supervised learning model used for classification or regression problems, based on the search for the hyperplane that is able to separate the different classes that make up the entire parameter or feature space [22]. In this particular case, we use SVM to perform a regression that estimates the VA of patients based on previously extracted biomarkers that constitute the feature set. Specifically, we use both the area of the FAZ in the SCP (in the 3 × 3 mm OCTA images) and the VD in each of the five zones of the grid (in the 3 × 3 and 6 × 6 mm OCTA images) in the SCP and the DCP. The process for estimating VA is mainly based on separating the total data set into two: training and testing. The training set is used to find a function that forms the hyperplane that relates the input values to the VA, the test set is used to quantify the error while trying to estimate new data with the function that was previously designed. A cross-validation 5-fold was used, which gives stability to the results even varying the training set.

The analysis of OCTA images from image input to VA estimation is performed in real-time.

### 2.5. Experiments

We performed two different experiments to validate the proposed methodology in RVO patients and to assess its capacity to estimate the visual acuity, first using only the area information of the FAZ obtained from the 3 × 3 mm OCTA images in the SCP and the 5 VD values obtained from the ETDRS grid (foveal, upper, nasal, temporal, lower) in the 3 × 3 and 6 × 6 mm OCTA images in the SCP, and second using the FAZ area obtained from the 3 × 3 mm OCTA images in the SCP, the 5 VD values obtained from the ETDRS grid in the 3 × 3 and 6 × 6 mm OCTA images in the SCP and the 5 VD values obtained from the ETDRS grid in the 3 × 3 and 6 × 6 mm OCTA images in the DCP.

**First experiment:** To assess the capacity of the proposed automatic method to estimate visual acuity.

**Second experiment:** To assess if we can estimate visual acuity more accurately by incorporating the depth information provided by the proposed method.

### 2.6. Statistical Analysis

All analyses were conducted using SPSS Statistics 20 (California State University, Los Angeles, CA, USA). The data are expressed as average ± standard deviation (SD). The BCVA was measured with the ETDRS acuity chart, and the decimal BCVA was converted to the logarithm of the minimal angle of resolution (logMAR) for the statistical analyses. The mean area of FAZ on the 3 × 3 mm images and the mean VD on the 3 × 3 and 6 × 6 mm images on the SCP were calculated and expressed as average ± SD, both for the semiautomatic system and the proposed automatic method measurements. The mean VD in the 3 × 3 and 6 × 6 mm images on the DCP were calculated for the data obtained from the automatic proposed method. A Pearson correlation analysis was performed to correlate the estimated BCVA using the VD data obtained with the Triton device and the manual FAZ, with the real BCVA of the patient, as well as the estimated BCVA using the proposed automatic method, with the real BCVA, and the estimated BCVA using the proposed method when considering the depth data, with the real BCVA. We calculated the mean squared error (MSE) of the BCVA estimation in each of the three proposed scenarios (i.e., when the manual and semiautomatic data from the device on the SCP were considered, when the automatic data from the novel proposed method on the SCP were considered, or when the automatic data from the proposed method including the information from the DCP were considered). Within the last proposed scenario and for deeper analysis, we divided the sample into four groups: treated, untreated, phakic, and pseudophakic patients, and we calculated the MSE of the BCVA estimation for each group. For this calculations, we used the decimal BCVA data because it follows a linear distribution, the SVM model estimates VA using a linear, rather than logarithmic, distribution. Results were considered statistically significant at *p* < 0.05. 

## 3. Results

Fifty eyes of 50 patients with RVO, 27 (54%) women and 23 (46%) men, were examined in this study. The mean age was 70.7 ± 11.296 years (range: 51–92 years). A total of 9 (18%) patients were diagnosed with central RVO and 41 (82%) with branch RVO. Forty-four (88%) patients presented ME at baseline and 6 (12%) patients did not have ME but did have macular ischemia. The 44 patients with baseline ME received intravitreal antiVEGF treatment and the remaining patients were followed-up without any treatment. Thirteen (26%) patients were pseudophakic and 37 (74%) patients were phakic. Baseline diseases and other clinical characteristics for the entire cohort are summarized in Table 1. 

Of the 50 patients included in the study, 35 contributed with three visits, two patients only had the first visit, and one patient only had the first and second visits, because of lack of follow-up (two deaths and one macular hole development, respectively). For 11 patients, we excluded the baseline visit because of macular hemorrhage causing a poor signal strength index, and in one patient, we excluded the second visit because one of the images was not correctly centered on the fovea and the data of inferior paracentral VD was not available. Finally, 133 visits (39 at baseline, 47 at 3 months, and 47 at the 6-month follow-up) were included in the subsequent analysis. 

The mean BCVA in eyes with RVO was 0.473 ± 0.389 at baseline, 0.324 ± 0.371 on the second visit, and 0.306 ± 0.441 on the third visit. 

As explained in Materials and Methods, we performed two different experiments to validate the proposed methodology in RVO patients.

### 3.1. First experiment: To Assess the Capacity of the Proposed Automatic Method to Estimate Visual Acuity

A preliminary analysis was performed to assess the correlation between the studied OCTA parameters (FAZ and VD) and the real VA. The VD was independently analyzed for each of the five zones of the ETDRS grid and for the global value (mean VD of the grid). As shown in Table 2, the obtained correlation coefficients were weak, suggesting this system is not acceptable for accurately estimating VA. As a result, we applied a regression system using SVM to estimate VA using the same parameters. For the VA estimation using SVM, both the area of the FAZ in the SCP (in the 3 × 3 mm OCTA images) and the VD in each of the five zones of the ETDRS grid in the SCP (in the 3 × 3 and 6 × 6 mm OCTA images) were used.

**Figure 3:**
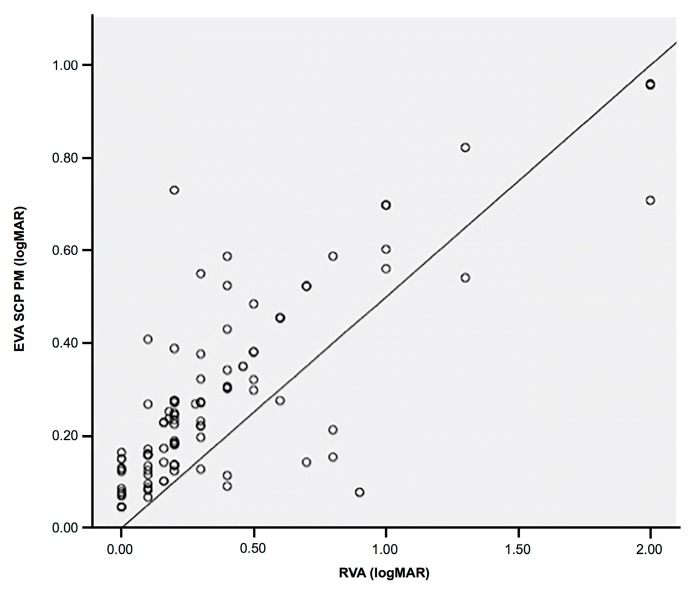
Dispersion diagram showing a linear positive correlation between the estimated visual acuity using the automatic foveal avascular zone and superficial capillary plexus vascular density data obtained using our software, and the real VA of the patient. EVA: estimated visual acuity, SCP: superficial capillary plexus, PM: proposed method, RVA: real visual acuity.

**Figure 4:**
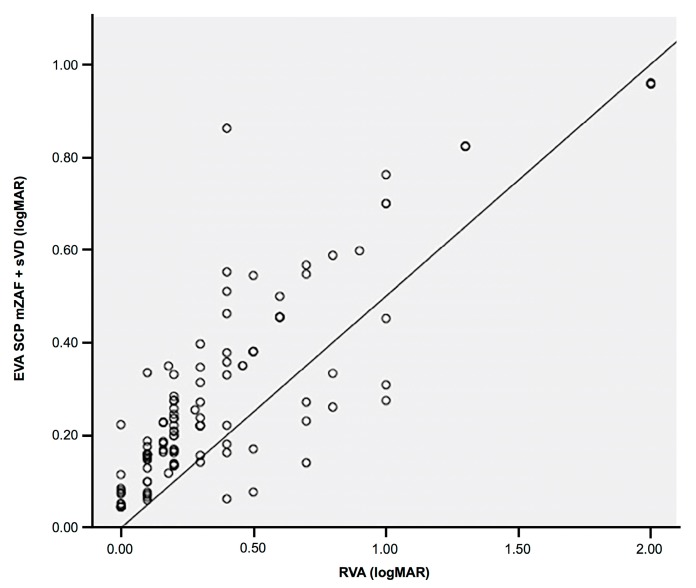
Dispersion diagram showing a linear positive correlation between the estimated visual acuity using the manual foveal avascular zone + device vascular density data on superficial capillary plexus, and the real visual acuity of the patient. EVA: estimated visual acuity, SCP: superficial capillary plexus, mFAZ: manual foveal avascular zone, sVD: semiautomatic vascular density, RVA: real visual acuity.

The VA estimated using the proposed method correlated with the real VA with a high correlation index of 0.833 (Table 3, Figure 3). As can be observed, it was less accurate in estimating VA compared with the VA estimation using the manual FAZ and the device VD when considering the data of the SCP (Table 3, Figure 4). The proposed methodology was able to estimate VA with an MSE of 0.093, while the Triton software with the manual FAZ produced an error of 0.064 at the decimal scale (Table 4). The results obtained in this section were extracted through the test phase of the algorithm.

### 3.2. Second Experiment: To Assess If We Can Estimate Visual Acuity More Accurately by Incorporating the Depth Information Provided by the Proposed Method

In addition to the information obtained from the SCP, we incorporated to the method the information obtained from the DCP. Specifically, the data used to perform the second experiment were: the area of the FAZ obtained from the 3 × 3 mm OCTA images in the SCP, the 5 VD values obtained from the ETDRS grid (foveal, upper, nasal, temporal, lower) in the 3 × 3 and 6 × 6 mm OCTA images in the SCP and the 5 VD values obtained from the ETDRS grid in the 3 × 3 and 6 × 6 mm OCTA images in the DCP.

**Figure 5:**
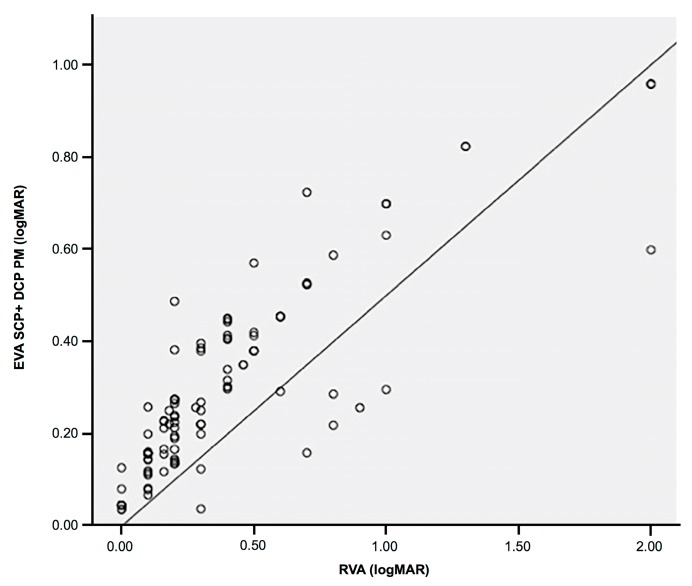
Dispersion diagram showing the most accurate linear positive correlation between the estimated visual acuity (VA) when deep capillary plexus information was integrated into the proposed automatic software and the real VA of the patient. EVA: estimated visual acuity, SCP: superficial capillary plexus, DCP: deep capillary plexus, PM: proposed method, RVA: real visual acuity.

When the depth information was integrated into the proposed automatic method, the results were much better, and our system was able to estimate the BCVA more accurately than when only using data of the SCP. The estimated VA when considering the VD of the DCP correlated with the real VA at a high correlation index of 0.869 (Table 5, Figure 5), generating a calculation error of only 0.055 (Table 6). The results obtained in Table 6 were extracted through the test phase of the algorithm.

To reduce possible biases in the results arising from differences that could exist between patients treated with intravitreal antiVEGF injections and untreated patients, and between phakic and pseudophakic patients, a more extensive analysis was performed for each of the above groups. In order to perform this analysis, it was necessary to enter all patients into the system algorithm, rather than just the test set, as the test set represented a negligible part of the total sample, and given that untreated patients and pseudophakic patients were a small percentage of the total sample (12% and 26%, respectively), using only the test set would greatly limit the results. Therefore, the following experiment was carried out using data from all patients for comparative purposes. As can be observed in Table 7, the results of the entire sample were better than those obtained by the test (MSE of 0.018 and 0.055, respectively). This is due to the fact that for this experiment, data were used that had previously been used to train the system. As we can see, the difference in the estimation of the VA between the group of treated and untreated was minimal. A slightly larger difference was observed between the group of phakic and pseudophakic patients.

Once it was demonstrated that the proposed method was applicable to RVO patients, we made an ultimate sub-experiment to demonstrate if it could be applied to healthy eyes. The proposed algorithm, which had only been trained with FAZ and VD data from OCTA images of pathological RVO eyes, was introduced with FAZ and VD data extracted from 3 × 3 and 6 × 6 mm OCTA images of ten healthy eyes, to try to estimate the VA in each case. The vascular parameters were the same as those described at the start of the second experiment of section Results and were automatically obtained through the methods proposed in section Material and Methods. This sample consists of 10 healthy eyes of 10 patients, five (50%) women and five (50%) men, of age greater than or equal to 18 years, without any past or present retinal disease, absence of refractive error greater than six diopters, and with enough transparency of ocular media to allow quality OCTA images. All the patients had a real VA of 1.000 in decimal scale. The proposed method estimated the VA with an MSE of 0.134 for healthy eyes (Table 8).

## 4. Discussion

The appearance of OCTA technology in the field of ophthalmology represented the arrival of a new era, although we are still realizing the great potential of this technology. The importance of this novel technology lies in the fact that the classic angiographic methods are invasive, as they require the intravenous injection of a contrast dye in order to obtain suitable images of retinal and choroid vascularization. Furthermore, there are concrete cases in which this procedure is not recommended or is prohibited (for example, in the case of pregnancy, renal insufficiency, or contrast dye allergy). Conversely, OCTA is a novel technique that, in addition to its demonstrated superiority in assessing the macular vascularization compared with FA techniques [4], has the main advantages of being noninvasive and less time-consuming than classic procedures. It is rapid enough to be used in every examination during diagnosis and follow-up. Considering the increased workload that exists in modern clinical practice, this is especially significant as it may allow for a faster workflow while maintaining high medical quality.

Furthermore, this new tool provides stratigraphic vascular details that have not been previously observed by standard fluorescein angiography, allowing us to independently visualize not only the superficial but also the deep capillary plexus and the choriocapillaris, such that we can better understand the pathophysiology and define the extent of the damage in increasingly prevalent retinal vascular injuries, such as RVO. Quantification of the extent of the damage at baseline and during follow-up can offer us new treatment approaches or additional information about treatment responses. 

In a recent study of 14 healthy and central serous retinopathy eyes, Wang et al. [6] showed that SS-OCTA is advantageous over SD-OCTA when analyzing vascular information from deeper tissues. For that reason, the DRI SS-OCTA Triton was selected to perform our RVO-based study.

In addition to systemic risk factors such as hypertension, hyperlipidemia, thrombofilia [23], diabetes mellitus, cerebral vascular stroke or cigarette smoking, the main ocular risk factors for RVO are glaucoma and ocular hypertension [24]. It has been previously demonstrated that macular vascular density (VD) in patients with glaucoma is significantly lower in both superficial and deep capillary plexus compared to healthy eyes [25]. Therefore, no patients diagnosed with glaucoma or ocular hypertension were included in the study, as it could have introduced a bias in VD results. Another source of bias in VD measurement has also been shown to be diurnal retinal flow variation. Baek et al. [26] recently used the same OCTA device that we used in this study and demonstrated that in healthy patients, macular VD shows statistically significant diurnal variability for the five zones of the ETDRS grid. However, in our study, all examinations were performed in the morning between 9 and 11 a.m.

Our study yielded two remarkable results: First, we developed an innovative computational model that is able to fully automatically estimate VA using only data of the FAZ area and the VD obtained from the 3 × 3 and 6 × 6 mm OCTA images of the SCP centered on the fovea without implementing any other information of the patient. 

It has been previously demonstrated that the area of the FAZ in RVO eyes is correlated with visual prognosis [8,9,10,11,12,13,27,28]. It has been reported that only the FAZ in the SCP correlates with visual acuity while the FAZ in the deep plexus does not show a significant correlation with visual acuity in RVO eyes [10,12,27]. However, Samara et al. [11] and Wons et al. [9] have demonstrated otherwise. Furthermore, the vascular density of the macula has shown to be another determining parameter of the visual outcome in RVO eyes [10,11,12,13].

Second, we demonstrated that the integration of DCP VD information into the proposed method resulted in even more accurate estimates of VA, and we obtained a higher correlation coefficient of 0.869.

OCTA technology offers us information about the retinal DCP, which is of special importance in RVO, considering that some authors have previously demonstrated the main damage of the deep capillary plexus (rather than the SCP) induced by this disease [14,15,16,17]. Coscas et al. [14] studied 54 RVO eyes, reporting that nonperfusion areas were mostly observed in the DCP (84%) rather than the SCP (59%), and they found a significant correlation with retinal peripheral ischemia on FA. Furthermore, Adhi et al. [15] prospectively analyzed 23 RVO eyes, showing a decrease in macular vascular perfusion in the DCP in all eyes with central and branch RVO, with a less frequent vascular decrease in the SCP in both groups. Similarly, Mastropasqua et al. [17] evaluated the macular VD in 60 RVO eyes with ME, 38 of which were analyzed before and after treatment with an intravitreal dexamethasone implant, assessing the correlation with visual acuity and microperimetry. They concluded that the whole retinal VD is reduced in RVO, with main involvement of the DCP, and no recovery was observed after intravitreal dexamethasone implant.

In a retrospective study of 33 RVO patients and 33 healthy controls, Kang et al. [10] demonstrated that the FAZ in the SCP, as well as the superficial and deep macular VD, were the only vascular parameters associated with the BCVA, results that were corroborated by our study. Winegarner et al. [28] retrospectively studied 35 subjects with central RVO after ME resolution following antiVEGF and found a significant correlation between the BCVA outcomes and both a wider FAZ area and a lower VD on the superficial and deep capillary plexus. Jung et al. [13] recently analyzed the macular vascularization in a cohort of 10 RVO patients to compare the single with the averaged 3 × 3 mm images, and concluded that the FAZ area and the VD on the SCP and DCP were correlated with the BCVA in both scan modalities. These conclusions strengthen the results of our study. Conversely, Mastropasqua et al. [17] did not find a significant correlation between VD and the functional visual parameters (visual acuity and microperimetry). However, in a recent study involving 65 RVO eyes, Seknazi et al. [29] also found a significant correlation between the superficial and deep VD and visual acuity, but not FAZ area. Unlike in our study, they only considered the VD of the 3 × 3 mm OCTA images centered on the fovea. Among all the vascular parameters in OCTA, Khodabandeh et al. [30] found that the flow in choriocapillaris was the parameter most strongly correlated with VA, but attributed it to the severity of the disease.

As has been shown, the studies published so far have mainly been focused on assessing whether a correlation exists between VA and the OCTA features in RVO. Unfortunately, none have managed to develop a fully automatic system able to rapidly obtain a highly accurate VA estimation from the macular OCTA images acquired during patient visits and only using data on the area of FAZ and VD. To our best knowledge, this study is the first attempt aimed at developing an automatic computational methodology to obtain functional information based only on OCTA images.

In addition to the proposed novel system to estimate the VA based on OCTA images, we also proposed, in this study, a fully automatic and robust method to measure the area of the FAZ and the macular VD data on the SCP and DCP.

As it is important to have time-efficient imaging devices to assess ophthalmological diseases, it is also essential to have automatic and objective tools that provide quantitative vascular information extracted from the retinal images. The DRI SS-OCTA Triton allows us to manually measure the FAZ area using tools provided by the device software. Ghasemi et al. [31] used another device to study the vascular changes in five central RVO eyes after a single intravitreal antiVEGF injection for ME eyes using a manual tool to outline the FAZ. In this respect, the intrapersonal and interpersonal variability of the manual FAZ identifications has to be considered as this introduces subjectivity into the calculation, in contrast to the objectivity and repeatability of the computational procedure that we have proposed [18]. 

The Triton software also gives us information about the superficial VD on the 3 × 3 and 6 × 6 mm OCTA images in each of the five zones of the ETDRS grid. Although this procedure is automatic in patients without macular damage, i.e., in which the FAZ is easily identifiable, in the frequent RVO cases where there are macular nonperfusion areas, this method incorrectly identifies FAZ, such that it becomes necessary to manually relocate the ETDRS grid to obtain vascular data. This results in the method being time-consuming and only semiautomatic. In addition, it does not provide quantitative information about the deep capillary plexus, which we have shown to be essential, especially in vascular occlusive diseases.

In the current study, we proposed a novel, fully automatic, objective, and time-efficient methodology for assessing different vascular parameters which are considered as prognostic biomarkers in RVO eyes. We used a metric based on a new application of the methodology previously validated by Díaz et al. [18] for healthy and diabetic patients, summarized in Materials and Methods, which we applied here to RVO OCTA images, and integrated an image processing technique to include the VD information. Finally, we implemented this information to estimate VA, based on the extracted parameters.

Considering only the data obtained from the SCP, our methodology showed a slightly less accurate VA estimation than when VD data were semiautomatically obtained from the device and a manual FAZ was used (MSE of 0.093 in the testing phase when applying our method compared to an error of 0.064 when applying the manual and semiautomatic method). Nevertheless, this is not a significant difference in terms of clinical practice, and considering that the existing method introduces subjectivity into the calculation, we consider that the difference is negligible. However, our study demonstrated that our method can automatically calculate the depth information—a determinant prognostic factor which is absent in the semiautomatic methodology. By integrating the depth information, our metrics demonstrated improved accuracy when estimating VA, providing a better VA estimation than when using the Triton superficial VD and manual FAZ data. In addition, once the system had been trained and when it was implemented using the SCP and DCP data of the whole sample, the results were even better, obtaining an MSE of 0.018, much more accurate than the MSE of 0.055 obtained in testing phase. 

Since the sample of patients with OVR was heterogeneous, in the sense that it included treated and untreated patients, as well as phakic and pseudophakic patients, we divided the sample into subgroups to analyze them in-depth to see if there were differences in the VA estimation results among them. The proposed method made a very accurate estimation of the VA, showing an MSE of only 0.019 for treated patients and 0.017 for untreated patients, with a minimum difference between both groups. Winegarner et al. [32] studied the changes in retinal microvasculature before and after 12 months of treatment with intravitreal antiVEGF injections in 48 subjects with RVO, demonstrating that SCP and DCP VD and FAZ did not change significantly after treatment. That is, in their study therapy with antiVEGF did not deteriorate or improve vascular flow in the macular area of patients with RVO. Ghasemi et al. [31] and Sellam et al. [33] concluded that the perfused macular microvasculature at baseline could be maintained for at least 12 months with anti-VEGF therapy in RVO eyes. In our study we have included 133 baseline and follow-up visits of patients with RVO. The proposed method is able to estimate the VA of the patient with high precision for both treated and untreated patients, so we can state that the measurement of vascular parameters (FAZ and VD in SCP and DCP) in OCTA images is accurate in both groups at baseline and in follow-up visits. Previous authors [31,32,33] show that these vascular parameters do not change after treatment with antiVEGF. This is not incompatible with our results since, whether or not the vascular parameters change during the follow-up, our system is capable of making precise measurements of these parameters and would be able to detect such changes. This is important in the follow-up of the patient with RVO, whose analysis using the automatic method proposed in this study could be interesting object of study in future works.

In the case of phakic and pseudophakic subgroups, we observed a very slight difference, which indicates that the transparency of means between both groups is comparable and must not significantly alter the results. However, in the pseudophakic group, the MSE for the VA prediction is higher. This can be explained by the small size of the group (13 patients, 26%) compared to the group of phakics (37 patients, 74%), in which there is a higher percentage of patients with extremely low VA, producing less accurate VA estimations. However, this difference is clinically insignificant, considering that we are working with a reference range of VA between 0 and 1 on the decimal scale.

Once it was proven that the proposed method was applicable to RVO eyes, a supplementary experiment was performed to demonstrate if it could be successfully performed in healthy patients, obtaining a VA estimation with a MSE of 0.134, compared with the previous MSE of 0.055 of the model, which had only been trained using pathological images. These results, although slightly worse, are equally satisfactory, since they allow the estimation of VA in healthy patients, even when the system had never trained with OCTA images of healthy eyes, making an assumable error. Hence, further training with a greater number of OCTA images of healthy eyes would probably provide more accurate results. However, clinically an MSE of 0.134 in the estimation of VA is an acceptable result.

Therefore, our methodology is reliable, has been validated for OCTA images of RVO eyes, and is even more precise when considering both the superficial and deep capillary plexus, outperforming the previous semiautomatic methodology. Furthermore, it only applies objective computational procedures to obtain vascular information from the OCTA images and uses a cross-validation 5-fold, which gives stability to the results even varying the training set. However, future studies must be performed to demonstrate the reproducibility of the technique.

In addition, the proposed method is fully automatic and less time-consuming, as it directly gives us complete information of the superficial and deep retinal layers from 3 × 3 and 6 × 6 mm OCTA images and estimates VA at each visit in real-time, without the intervention of the human factor in any step.

In an interesting study, Tsuboi et al. [34] analyzed 20 branch RVO eyes in one single visit, and proposed the VD difference between the superficial and deep capillary plexus as a new biomarker of ME recurrence and used an image processing technique to visualize the isolated vessels using OCTA. The main difference with the method proposed in our study is that we could validate our metrics on the SCP by correlating them with the estimated VA obtained when using the device data, as well as demonstrating a more accurate estimation of VA. Furthermore, our metrics included the automatic calculation not only of the VD but also FAZ, so the proposed method is more complete.

Our study has some limitations. First, the treated patients received two different antiVEGF treatments (ranibizumab or aflibercept). Second, taking into account that the proposed methodology is based on an artificial intelligence system, it is understandable that the VA estimation was more inaccurate in the few cases of extremely low VA, due to the insufficient number of available image cases of this type from which to learn. However, this degree of extreme vision loss is uncommon in RVO patients, so does not represent a limitation for the main cases of RVO. Third, the OCTA device has intrinsic limitations. The SS-OCTA instrument software is a depth-resolved imaging modality that improves penetration through the ocular tissues as well as optical opacities [5]. However, we could not obtain high-quality images at baseline in patients who presented dense hemorrhages in the macular area, due to blood opacity, and these visits had to be excluded from the study. Furthermore, patients with poor fixation produced motion artifacts in the OCTA images, and those could not be included. Ultimately, the Topcon device does not give quantitative information about the deep structures, making it impossible to validate our metrics in the deep plexus with the automatic method in this study, as we could not compare them with the semiautomatic system. However, the results obtained in this study are in accordance with previously reported imaging and histopathological retinal findings on RVO, in which the influence of main damage of the DCP on visual function was demonstrated. 

The clinical utility of the proposed method in assessing vascular evolution during RVO patient follow-up and evaluating responses to different treatments, as well as accurately estimating VA, may provide the basis for useful applications in the field of telemedicine and save many healthcare resources. In this sense, its application in other retinal vascular diseases needs to be studied in future research. As a next step to be addressed in future studies, the system could be taught to recognize other patterns in retinal vascular layers and the choriocapillaris and, therefore, use them as biomarkers of visual prognosis and integrate them into this innovative automatic method for estimating VA. In addition, building a more extensive database using a larger sample group and further follow-up visits would make it possible to develop an automatic system to not only estimate but also predict VA, which is another potential future line of investigation. 

## 5. Conclusions

We have developed a novel method to estimate VA based only on the OCTA information of RVO patients. The proposed method improves upon the results obtained when using device data and manual FAZ measurements, and it is fully automatic, less time-consuming, objective, and more accurate at estimating VA and, therefore, has greater validity. In addition, it integrates depth information, which is lacking in the existing device.

## Figures and Tables

**Table 1:** Baseline diseases and other clinical characteristics for the entire cohort.

Patient Characteristics	No. (%)
**Hypertension**	37 (74)
**Hyperlipidemia**	28 (56)
**Diabetes mellitus**	5 (10)
**Vascular cerebral stroke**	2 (4)
**Smoker**	3 (6)
**Former smoker**	6 (12)
**Antithrombotic drugs**	11 (22)
**Anticoagulant drugs**	2 (4)

No: number.

**Table 2:** Correlation coefficients obtained by correlating the area of the foveal avascular zone, the vascular densities in the five zones of the Early Treatment Diabetic Retinopathy Study (ETDRS) grid and the global vascular density, with the real visual acuity (VA).

*r*	3 × 3 mm SCP	3 × 3 mm DCP	6 × 6 mm SCP	6 × 6 mm DCP
**FAZ**	0.125	0.021	-	-
**Foveal**	0.351	0.352	0.014	0.247
**Upper**	0.128	0.070	0.071	0.186
**Nasal**	0.097	0.130	0.112	0.290
**Temporal**	0.016	0.060	0.146	0.313
**Lower**	0.125	0.193	0.295	0.271
**Global**	0.149	0.337	0.246	0.446

*r*: Pearson correlation index, SCP: superficial capillary plexus, DCP: deep capillary plexus, FAZ: foveal avascular zone.

**Table 3:** Correlation coefficients obtained from correlating the estimated visual acuity using the manual foveal avascular zone (FAZ) + device vascular density (VD) data on superficial capillary plexus (SCP) and the estimated visual acuity using the automatic FAZ and SCP VD data.

*r*, *p*	VA, Manual FAZ + Device VD (SCP)	VA, Our Software (SCP)
Real VA	0.855, 0.000	0.833, 0.000

*r*: Pearson correlation index, VA: visual acuity, FAZ: foveal avascular zone, VD: vascular density, SCP: superficial capillary plexus.

**Table 4:** Mean squared error of the visual acuity estimation when using the device vascular density (VD) in the superficial capillary plexus (SCP) and the manual foveal avascular zone (FAZ) data, and the automatic FAZ and SCP VD data obtained using our software.

Data Source	MSE
Device VD + manual FAZ (SCP)	0.064
Our software (SCP)	0.093

MSE: mean squared error, VD: vascular density, FAZ: foveal avascular zone, SCP: superficial capillary plexus.

**Table 5:** Correlation coefficients obtained from correlating the estimated visual acuity (VA) using the manual foveal avascular zone (FAZ) + device vascular density (VD) data on superficial capillary plexus (SCP) and the estimated VA using the automatic FAZ and SCP + deep capillary plexus VD data.

*r*, *p*	VA, Manual FAZ + Device VD (SCP)	VA, Our Software (SCP + DCP)
Real VA	0.855, 0.000	0.869, 0.000

*r*: Pearson correlation index, DCP: deep capillary plexus.

**Table 6:** Mean squared error of the visual acuity estimation when using the device vascular density (VD) on the superficial capillary plexus (SCP) and the manual foveal avascular zone (FAZ) data, and the automatic FAZ, SCP, and deep capillary plexus VD data obtained using our software.

Data Source	MSE
Device VD + manual FAZ (SCP)	0.064
Our software (SCP + DCP)	0.055

MSE: mean squared error, DCP: deep capillary plexus.

**Table 7:** Mean squared error of the visual acuity estimation when using the automatic area of the foveal avascular zone (FAZ), superficial capillary plexus vascular density (VD) data and deep capillary plexus VD data obtained using our software for each group and for the entire sample.

Our Software (SCP + DCP)	MSE
**Treated**	0.019
**Untreated**	0.017
**Phakic**	0.017
**Pseudophakic**	0.025
**Entire sample**	0.018

SCP: superficial capillary plexus, DCP: deep capillary plexus, MSE: mean squared error.

**Table 8:** Mean squared error of the visual acuity estimation when using the automatic area of the foveal avascular zone (FAZ), superficial capillary plexus vascular density (VD) data and deep capillary plexus VD data obtained using our software for healthy patients.

Our Software (SCP + DCP)	MSE
**Healthy eyes**	0.134

SCP: superficial capillary plexus, DCP: deep capillary plexus, MSE: mean squared error.
